# CD39 and CD326 Are Bona Fide Markers of Murine and Human Plasma Cells and Identify a Bone Marrow Specific Plasma Cell Subpopulation in Lupus

**DOI:** 10.3389/fimmu.2022.873217

**Published:** 2022-04-08

**Authors:** Van Duc Dang, Elodie Mohr, Franziska Szelinski, Tuan Anh Le, Jacob Ritter, Timo Hinnenthal, Ana-Luisa Stefanski, Eva Schrezenmeier, Soeren Ocvirk, Christian Hipfl, Sebastian Hardt, Qingyu Cheng, Falk Hiepe, Max Löhning, Thomas Dörner, Andreia C. Lino

**Affiliations:** ^1^ Deutsches Rheuma-Forschungszentrum, A Leibniz Institute, Berlin, Germany; ^2^ Department of Rheumatology and Clinical Immunology, Charité Universitätsmedizin Berlin, Berlin, Germany; ^3^ Faculty of Biology, VNU University of Science, Vietnam National University, Hanoi, Vietnam; ^4^ Berlin Institute of Health (BIH), Berlin, Germany; ^5^ Department of Nephrology and Medical Intensive Care, Charité-Universitätsmedizin Berlin, Corporate Member of Freie Universität Berlin and Humboldt-Universität zu Berlin, Berlin, Germany; ^6^ Intestinal Microbiology Research Group, Department of Molecular Toxicology, German Institute of Human Nutrition Potsdam-Rehbruecke, Nuthetal, Germany; ^7^ Centre for Musculoskeletal Surgery, Department of Orthopedics, Charité Universitätsmedizin Berlin, Berlin, Germany

**Keywords:** plasma cells, plasma cell markers, CD39, CD130, CD81, CD326, LAG-3, SLE

## Abstract

Antibody-secreting cells (ASCs) contribute to immunity through production of antibodies and cytokines. Identification of specific markers of ASC would allow selective targeting of these cells in several disease contexts. Here, we performed an unbiased, large-scale protein screening, and identified twelve new molecules that are specifically expressed by murine ASCs. Expression of these markers, particularly CD39, CD81, CD130, and CD326, is stable and offers an improved resolution for ASC identification. We accessed their expression in germ-free conditions and in T cell deficient mice, showing that at least in part their expression is controlled by microbial- and T cell-derived signals. Further analysis of lupus mice revealed the presence of a subpopulation of LAG-3^–^ plasma cells, co-expressing high amounts of CD39 and CD326 in the bone marrow. This population was IgM^+^ and correlated with IgM anti-dsDNA autoantibodies in sera. Importantly, we found that CD39, CD81, CD130, and CD326 are also expressed by human peripheral blood and bone marrow ASCs. Our data provide innovative insights into ASC biology and function in mice and human, and identify an intriguing BM specific CD39^++^CD326^++^ ASC subpopulation in autoimmunity.

## Introduction

Antibody secreting cells (ASCs) are terminally differentiated B cells specialized for antibody production. ASCs have been shown to be involved in the pathogenesis of autoimmune diseases through production of autoantibodies and pro-inflammatory cytokines. Nevertheless, recent studies in mice showed that certain ASC subsets can act as negative regulators of immunity, through production of the anti-inflammatory cytokines IL-10 and IL-35 (1–3). Different subsets of ASCs have been described in mouse and human. For instance, a subset of CD19^-^ human bone marrow ASCs expressing high levels of programmed cell death protein-1 was recently reported (4). Collectively, ASCs form a heterogeneous population containing several subpopulations with different functions. These observations raised the possibility to selectively target pathogenic subpopulations in the context of autoimmunity, allergenic and malignant diseases. Such targeting is often limited due to the lack of specific markers and heterogeneity of surface molecule expression in various organs and diseases.

ASC differentiation is controlled by the expression of key transcription factors, including BLIMP-1, IRF4, and XBP-1 in both mouse and human (5, 6). *prdm1*eGFP mice are widely used to study various aspects of ASCs biology, such as their origin, phenotype, differentiation process, and function. Although the value of genetic mouse models has permitted deeper insights into ASC biology, such tool is not available for human studies. Therefore, new strategies to identify ASCs independently of BLIMP-1 or other transcription factors are necessary. Several surface ASC markers have been previously identified, including CD138 (Syndecan-1), CD267 (TACI or TNFRSF13B), CD98, CD319 (SLAM7), and SCA-1 (Ly6A/E) (6). However, the expression of these surface molecules is not restricted to ASCs. For example, CD138 is also expressed by a number of other cell types such as epithelial cells, fibroblasts, vascular smooth muscle, and endothelial cells (7, 8). CD267, a protein belonging to the BAFFR family protein that is required for maturation, maintenance and survival of B and ASCs, is also expressed by other B cell subsets and T cells (9–11). CD98, also known as LAT1, is expressed in several immune and non-immune cell types in both mouse and human (12–18). CD319 was found to be expressed by several immune cell types in human (19, 20) and SCA-1 is expressed by hematopoietic progenitor cells and is induced in many immune cell types upon type I interferon or TNF stimulation (6, 21, 22). Thus, specific markers of ASCs are still missing, in particular selective markers of pathogenic ASCs that could serve as selective therapeutic target. Moreover, many surface markers defined in murine ASCs are not relevant in human ASCs, thereby limiting the possibility of translation across species.

Here, we report 12 new surface molecules that can be used to study ASC biology, especially CD39 (NTPDase1), CD81 (TAPA-1), CD326 (EpCAM), and CD130 (gp130 or Oncostatin M Receptor) that can be used to identify mouse and human ASCs. These markers provide an improved resolution for ASC identification in several lymphoid organs. Furthermore, we made use of mice raised in germ free conditions, T cell deficient, aged mice and mice with systemic autoimmunity to investigate the signals controlling the expression of these new markers, revealing these ASC’s origin, differentiation processes and functions under various conditions. Interestingly, increased expression of CD39 and CD326 identifies a BM specific subpopulation of ASCs in SLE mice. Our results open new avenues to consider CD39, CD326, CD130, and CD81 as potential therapeutic targets of plasma cells (PCs) in autoimmune diseases.

## Materials and Methods

### Mice

C57BL/6, *prdm1*eGFP, *Tcrβδ* ^-/-^, *Sle123* (all C57BL/6 background) mice were bred under specific pathogen-free conditions at the DRFZ and Charité Universitätsmedizin, Berlin, Germany. C57BL/6 mice were also bred under GF or SPF conditions at the German Institute of Human Nutrition, Potsdam, Germany. *Sle123* mice were evaluated for proteinuria twice weekly (Multistick, Siemens) starting from week 24 onwards. *Sle123* mice were considered to be sick and sacrificed for analysis when proteinuria >100mg/dL. Age-matched WT, healthy age-matched or young *Sle123* mice (9-12 weeks old) without proteinuria were included as healthy control mice. Mice were between 10-20 weeks old, unless otherwise stated. Gender-matched mice were used.

### Human Peripheral Blood and Bone Marrow

Human peripheral blood was collected from 12 HDs and six SLE patients. Human bone marrow (BM) was obtained from nine patients undergoing total hip arthroplasty.

### Antibodies

Murine specific monoclonal antibodies: CD3 (clone 17A2), CD11a (clone M17/4), CD18 (clone M18/2), CD16/CD32 (Fc Block, clone 2.4G2), CD19 (clone 6D5), CD39 (clone Duha59), CD44 (clone IM7), CD47 (clone miap301), CD49d (clone R1-2), CD54 (clone YN1/1.7.4), CD81 (clone Eat-2), CD98 (clone RL388), CD130 (clone 4H1B35), CD138 (clone 281-2), CD155 (clone TX56), CD172a (clone P84), CD205 (clone NLDC- 145), CD229 (clone Ly9ab3), CD267 (clone 8F10), CD274 (clone 10F.9G2), CD319 (clone 4G2), CD326 (clone G8.8), IgA (clone mA-6E1), IgM (clone RMM-1), LAG-3 (Clone eBioC9B7W), SCA-1 (clone D7) and human specific monoclonal antibodies: CD3 (clone UCHT1), CD14 (clone M5E2), CD19 (clone SJ25C1), CD20 (clone 2H7), CD27 (clone L128), CD39 (clone A1), CD81 (clone 5A6), CD130 (clone 2E1B02), CD138 (clone MI15), and CD326 (clone 9C4) were purchased from BioLegend, Miltenyi Biotec, BD Biosciences, Thermo Fisher or produced in house. LEGENDScreen™ Mouse PE Kit was purchased from BioLegend.

### NP-KLH Immunization

C57BL/6 mice were immunized intraperitoneally with 200µg of NP-KLH precipitated in alum and analyzed 7 days after immunization. NP-reactive ASCs were detected using fluorochrome-conjugated NP.

### Murine Cell Isolation and Flow Cytometry

Cell isolation and flow cytometry were performed as previously reported (1, 23). Briefly, BM cells were flushed from femur and tibia using a 1-ml syringe with a 26G needle in PBS supplemented with 0.5% (w/v) bovine serum albumin (BSA) (PBS/BSA). Spleen and mesenteric lymph node (mLN) were mashed in a Petri dish with PBS/BSA, passed through a 70-µm cell strainer (BD Biosciences), and then transferred into a 15-ml tube using a 25G needle to obtain single cell suspension. For isolation of kidney cells, the kidneys were minced using a scalpel and manually crushed on a 70-µm cell strainer using a syringe plunger, and then transferred into a 15-ml tube using a 25G needle. Collagenase D (Sigma) was used to isolate splenocytes in some experiments, in these cases each spleen was cut into two equal pieces, each piece of spleen was then minced separately using a scalpel and transferred into 4ml of serum-free RPMI (Life Technologies) supplemented or not with 1mg/ml collagenase D and incubated at 37°C for 30 minutes. Non-digested tissue pieces were also crushed on 70-µm cell strainer using a syringe plunger, and subsequently transferred into a 15-ml tube using a 25G needle to obtain single cell suspension. Bone marrow, spleen, and kidney red blood cells were lysed using red blood cell lysis buffer (Sigma). After lysis, the cells were resuspended in PBS/BSA and counted using MACSQuant ^®^ Flow Cytometer (Miltenyi Biotec). Prior to staining, the cells were blocked with anti-Fc receptor antibody (clone 2.4G2) for 15 minutes and then incubated with fluorochrome- and/or biotin-conjugated antibodies against surface molecules for 20 minutes. Biotinylated antibodies were subsequently stained with fluorochrome-conjugated Streptavidin for 15 minutes. For the screening experiments, surface-stained cells were then incubated another 15 minutes with PE-conjugated screening antibody (LEGENDScreen™ Mouse PE Kit, BioLegends). For the kinetic experiment, BM cells were stained at 30 minutes, 2, 4 and 6 hours after red blood cell lysis. Dead cells were excluded using propidium iodide. Stained cells were measured by BD FACSymphony or BD LSRFortessa.

### Human Mononuclear Cell Isolation and Flow Cytometry

BM mononuclear cells were isolated as previously reported (24). Briefly, fresh BM samples were physically minced in PBS/BSA/EDTA (Miltenyi Biotec) and subsequently filtered with 70-µm cell strainer. BM mononuclear cells were obtained by density gradient centrifugation using Ficoll-Paque PLUS. BM mononuclear cells were collected and washed twice with PBS/BSA/EDTA.

Human peripheral blood mononuclear cells were isolated from EDTA-anticoagulated whole blood by Ficoll density gradient centrifugation. BM (30x10^6^) and peripheral blood (PB) mononuclear (20x10^6^) cells were initially labelled with live/dead dye (Blue fluorescent reactive dye, Molecular Probes Invitrogen) in PBS according to the manufacturer’s instructions and washed with PBS. Labelled cells were blocked with FcR blocking reagent (Miltenyi Biotec) for 5 min. Cells were then stained for surface molecules with fluorochrome-conjugated antibodies for 20 minutes. Stained cells were measured using a BD LSRFortessa.

### Detection of Anti-dsDNA Antibodies by ELISAs

Mice blood was collected postmortem by cardiac puncture, and stored at 4°C for 8–12 hours in 1.5ml microtubes. The tubes were centrifuged at 4°C for 10 min at 13000rpm and the sera were transferred into new microtubes and stored at –20°C until analysis.

Anti-dsDNA IgG and IgM antibodies were measured by ELISA as previously described (25). Briefly, 96-well polystyrene plates were pre-coated with 10µg/ml methylated BSA (Sigma-Aldrich) in PBS for 3 hours at 37°C. The plates were washed with PBS and coated with calf thymus dsDNA (10µg/ml in PBS; Sigma Aldrich) for 3 hours at 37°C. The plates were washed with PBS and blocked with PBS/BSA (3%, w/v) (Sigma-Aldrich) overnight at 4°C, and washed with deionized water. One hundred microliters of 5-fold serial dilutions of each serum were prepared in PBS/BSA (1%, w/v) to obtain 1:20 to 1:62500 dilutions, and were deposited on the plate. After 2 hour incubation at 37°C, the plates were washed with PBS 0.05% (v/v) Tween-20 (Sigma). Next, 100µl of either biotin-conjugated anti-mouse IgG or IgM (0.5µg/ml; Southern Biotech) were added to the wells and incubated for 1 hour at 37°C. The plates were washed with deionized water and incubated with 100µl of ExtrAvidin^®^-Peroxidase (Sigma Aldrich) at a 1:5000 dilution in PBS/BSA (1%, w/v) for 30 min at 37°C. After washing with deionized water, 50µl of Tetramethylbenzidine (TMB) substrate solution (Thermo Scientific) were added to the wells. The reaction was stopped with 50µl of 2M sulfuric acid and read at 450nm on a spectrophotometer (SpectraMax Plus 384, Molecular Devices, USA).

Relative serum anti-dsDNA antibody titer was calculated by plotting the dilution factors of each serum sample in logarithm scale against the absorbance. All curves were drawn on the same graph, and an absorbance cut off value was decided, which crossed the curves in the linear regions of the majority of the curve. The dilutions at cut off values were read on the x axis and gave the relative dsDNA antibody titers of the samples.

### Data Analysis and Statistics

Flow cytometry data were analyzed with FlowJo™ software (version 10.7.1, TreeStar). Quantification and statistical analysis were performed using GraphPad Prism 9. Groups were compared using two-way ANOVA, unpaired t test or multiple unpaired t test, as indicated in figure legends. Considered p-values were: ^ns^p > 0.05; ^∗^p < 0.05; ^∗∗^p < 0.01; ^∗∗∗^p < 0.001; ^∗∗∗∗^p < 0.0001. Correlation matrices were generated using R corrplot package.

## Results

### ASCs in Naive Mice Express CD39, CD229, CD81, CD326, CD54, CD130, CD49d, CD172a, CD274, CD155, CD205, and CD47

**Figure 1:**
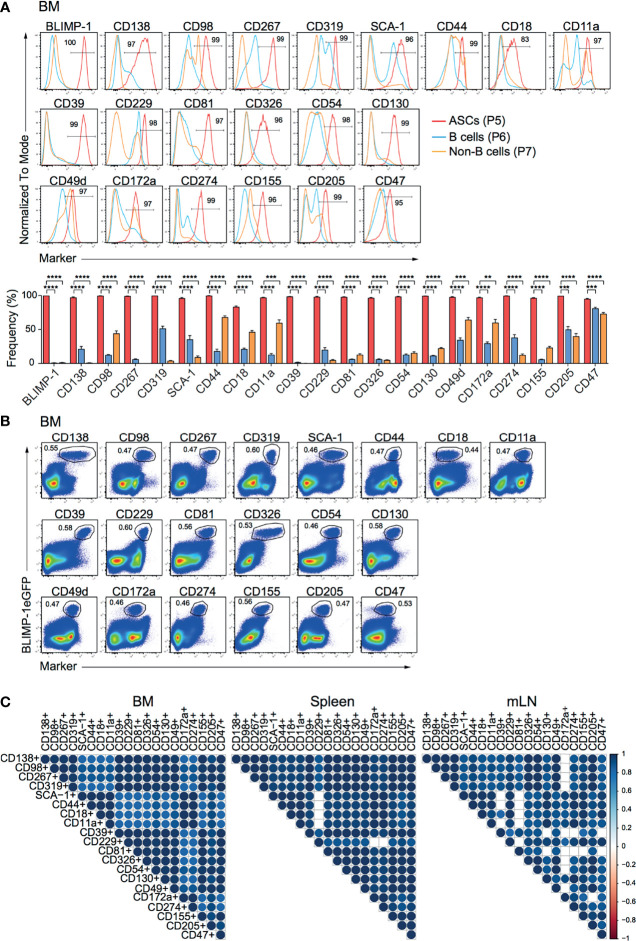
ASCs in naive mice express CD39, CD229, CD81, CD326, CD54, CD130, CD49d, CD172a, CD274, CD155, CD205, and CD47. Analyses were performed with naive *prdm1*eGFP mice. **(A)** Representative histograms (top, frequencies of ASCs are indicated) and frequencies (bottom) showing surface expression level of indicated markers by BM CD138^+^BLIMP-1eGFP^+^ ASCs, CD19^+^CD138^-^BLIMP-1eGFP^-^ B cells and CD19^-^CD138^-^BLIMP-1eGFP^-^ cells. Populations were gated as indicated in **Supplementary Figure 1A**. Groups were compared using two-way ANOVA with the Geisser-Greenhouse correction followed by Dunnett’s multiple comparisons test. Data represent mean ± SEM (^∗∗^p < 0.01, ^∗∗∗^p < 0.001, ^∗∗∗∗^p < 0.0001). **(B)** Representative FACS plots demonstrating the co-expression of BLIMP-1eGFP with indicated markers in BM (populations were gated on live cells). **(C)** Spearman correlation matrix of ASCs populations (frequencies) defined by co-expression of BLIMP-1eGFP and the indicated marker as shown in Panel **(B)** (BM), **Supplementary Figure 1D** (spleen) and **Supplementary Figure 1E** (mLN). Blue color indicates positive correlation with color intensity and size of the circle representing correlation coefficients. Only correlations with p<0.05 are depicted. Data shown were derived from two independent experiments (n=6 mice).

To identify new makers expressed by ASCs, we used splenocytes and BM cells isolated from naive *prdm1*eGFP reporter mice to screen 255 surface molecules (LEGENDScreen™, BioLegend, Inc.) by flow cytometry. ASCs identified as BLIMP-1eGFP^+^CD138^+^ were compared with B and non-B cells, identified as CD19^+^BLIMP-1eGFP^-^CD138^-^ and CD19^-^BLIMP-1eGFP^-^CD138^-^ cells, respectively (**Supplementary Figure 1A**). This strategy yielded 20 surface molecules, of which the high expression by ASCs from BM, spleen, and mLN was confirmed by further flow cytometric analysis (**Figure 1A**; **Supplementary Figures 1B, C**). Among these surface molecules, CD138, CD98, CD267, CD319, SCA-1, CD44, CD18 (Integrin β2), and CD11a (Integrin α-L) were found consistent with earlier reports for ASCs (6, 26). Most importantly, certain surface molecules, such as CD39, CD229 (Ly-9), CD81, CD326, CD54 (ICAM-1), CD130, CD49d (Integrin α4), CD172a (SIRPα), CD274 (PD-L1), CD155, CD205 (DEC-205), and CD47 were identified and have not been clearly reported as ASCs markers before. Importantly, flow cytometry clearly confirmed the co-expression with BLIMP-1eGFP^hi^ cells in BM (**Figure 1B** **)**, spleen **(** **Supplementary Figure 1D**) and mLN (**Supplementary Figure 1E**). To evaluate the co-expression of BLIMP-1eGFP and the candidate markers with the BLIMP-1eGFP^+^CD138^+^ population, we performed a Spearman correlation analysis. The majority of the populations defined by the new markers showed positive correlation (r > 0.94) with BLIMP-1eGFP^+^CD138^+^, confirming the co-expression of these molecules by the same ASCs (**Figure 1C**). Thus, we concluded that differential expression of CD39, CD229, CD81, CD326, CD54, CD130, CD49d, CD172a, CD274, CD155, CD205, or CD47 together with BLIMP-1eGFP can be used to identify ASCs independently of CD138 in lymphoid organs of naive mice.

### ASCs in Naive Mice Are Reliably Identified by the Expression of CD39, CD81, CD326, and CD130

**Figure 2:**
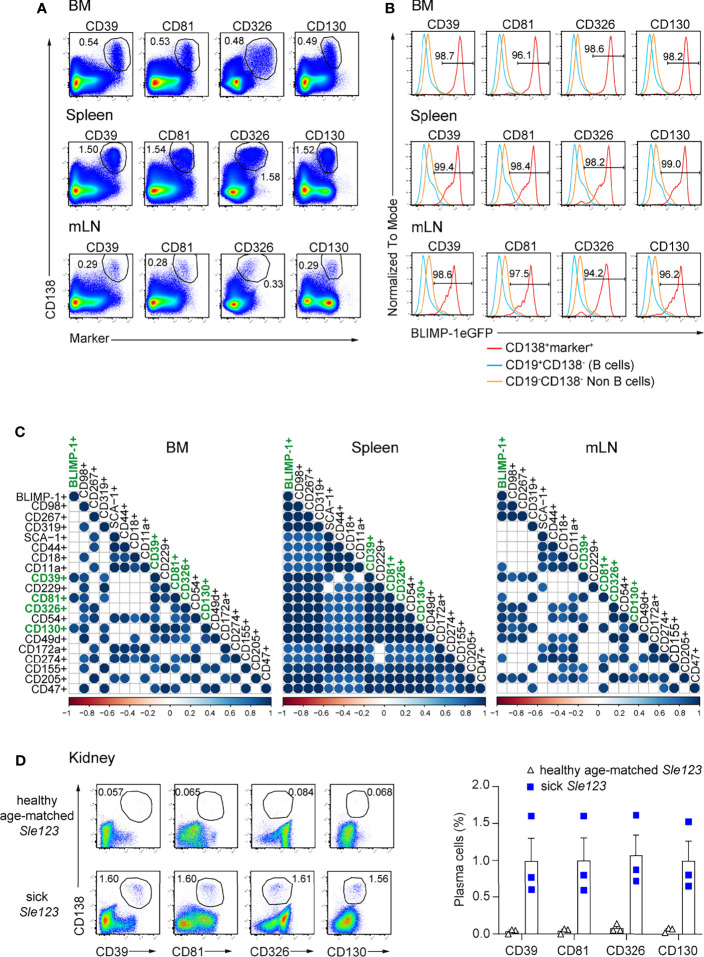
ASCs in naive mice are reliably identified by surface expression of CD39, CD81, CD326, and CD130. Analyses were performed with naive *prdm1*eGFP mice, unless indicated otherwise. **(A)** Representative FACS plots show the co-expression of CD138 with indicated markers on ASCs from BM, spleen, and mLN. The populations were gated on live cells. **(B)** Representative histograms showing frequencies of BLIMP-1eGFP^+^ cells within ASC populations as gated in **(A)**, CD19^+^CD138^-^ B cells and CD19^-^CD138^-^ non-B cells obtained from BM, spleen and mLN. **(C)** Spearman correlation matrix of ASCs populations (frequencies) defined by co-expression of CD138 and the indicated marker as shown in Panel **(A)** and **Supplementary Figure 2**. Positive correlation is displayed in blue with color intensity and size of circle reflecting correlation coefficients. Only correlations with p<0.01 are shown. **(A, B)** FACS plots and FACS histograms are representative of two independent experiments (n=6 mice). **(C)** The data were from two independent experiments (n=6 mice). **(D)** Representative FACS plots (left) and frequencies (right) of ASCs defined by co-expression of CD138 and the indicated marker in kidneys from sick (proteinuria >300mg/dL) and healthy age-matched *Sle123* mice (n=3 mice/group). The populations were gated on live cells. Data show mean ± SEM.

Next, we addressed whether the above identified markers can be used to identify ASCs independently of BLIMP-1. Thus, we first co-stained each marker with CD138, a bona fide ASC identifier. ASC populations co-expressing CD138 and each of the markers could be clearly identified in BM, spleen, and mLN. CD39, CD81, CD326, and CD130 provided an improved resolution compared other markers (**Figure 2A**; **Supplementary Figure 2**). Then, we asked whether double positive populations co-expressing CD138 and each marker would include only ASCs in lymphoid organs. To address this question, we quantified the frequencies of BLIMP-1-expressing cells within these populations. Most of these cells expressed BLIMP-1eGFP^+^ in all tested organs, except for a lower frequency in CD138^+^CD18^+^ cells, which contained higher proportions of non-BLIMP-1eGFP^+^ ASCs in both BM and mLN, as did CD138^+^CD319^+^ and CD138^+^CD49d^+^ cells in BM (**Figure 2B**). Therefore, these markers identified almost exclusively ASCs. Finally, we used a Spearman correlation matrix to evaluate which surface molecules would perform better than the others as ASCs identifiers in BM, spleen, and mLN, respectively. Most notably, only CD138^+^CD39^+^, CD138^+^CD81^+^, CD138^+^CD130^+^ populations exhibited a significant correlation with CD138^+^BLIMP-1eGFP^+^ population in BM when a p-value cutoff of 0.01 was applied (**Figure 2C**). CD326 was also significantly co-expressed when a p-value of 0.05 was considered. Of note, combinations of CD138 with CD98, CD267, CD319, CD39, CD229, CD81, CD326, CD54, CD130, CD49d, CD155, CD205, or CD47 allowed the identification of ASCs in spleen, whereas CD98, CD267, CD39, CD326, CD54, CD130, CD49d, and CD205 identified ASC in mLN (**Figure 2C**). Thus, CD39, CD81, and CD130 followed by CD326 are the best molecular ASC identifiers.

### CD39, CD81, CD326, and CD130 Expression Identifies Kidney-Resident ASCs in Lupus Mice

ASCs infiltrating in the kidney medulla of patients with SLE (systemic lupus erythematosus) correlate with kidney inflammation and disease severity (27). In animal models of lupus such as NZB/W F1 and MRL/lpr mice, ASCs infiltrate the kidneys and influence disease severity (27–29). Thus, we tested whether the herein identified markers can serve to quantify kidney-resident ASCs in mice with lupus. To do so, we analyzed mice of the B6.*Sle123* triple congenic strain, in which *Sle1* locus mediates the loss of immune tolerance; *Sle2* locus results in hyperactivity of B cells, and *Sle3* locus results in reduced CD4^+^ T cell apoptosis. B6.*Sle123* mice develop highly penetrant severe systemic autoimmunity and fatal glomerulonephritis, beginning at 6 months of age (30–32). Here, we analyzed 8 months old mice with established proteinuria (> 300 mg/dL, sick) and age-matched mice of the same strain in which proteinuria was absent (healthy). We found that co-expression of CD138 and CD39, CD81, CD326, and CD130 identified kidney-resident ASCs in sick *Sle123* mice while this ASC population was absent in proteinuria negative age-matched *Sle123* controls (**Figure 2D**). We conclude that CD39, CD81, CD326, and CD130 mark ASCs in kidneys of murine lupus nephritis.

### Stable Expression of CD39, CD81, CD326, and CD130 Allows Reliable Identification of BM and Tissue-Resident ASCs

**Figure 3:**
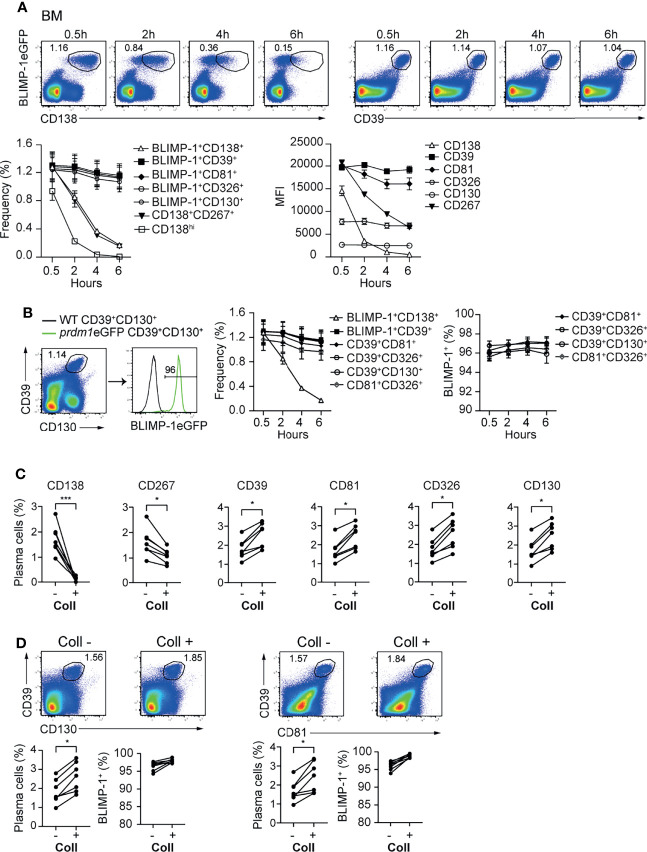
CD39, CD81, CD130, and CD326 expression is stable and provides improved resolution for ASC identification. Analyses were performed with naive *prdm1*eGFP mice, unless indicated otherwise. **(A)** Representative FACS plots (top) showing the co-expression of BLIMP-1eGFP with CD138 or CD39 in BM at indicated time points after BM single cell suspension preparation. The populations were gated on CD3^-^live cells. Results are representative of two independent experiments (n=7 mice). The graphs show the frequency of detected ASC (left) (gated according to top FACS plots and **Supplementary Figure 3B**) and MFI of indicated marker (right) in BLIMP-1eGFP^+^CD39^+^ ASCs (gated according to top right FACS plots). **(B)** Representative FACS plot demonstrating that ASCs gated based on the dual expression of CD39 and CD130 (left) are BLIMP-1eGFP^+^ (right). The graphs show the frequency of ASCs identified as BLIMP-1eGFP^+^CD138^+^, BLIMP-1eGFP^+^CD39^+^ (shown in A), CD39^+^CD130^+^ (shown in B), CD39^+^CD81^+^, CD39^+^CD326^+^ and CD81^+^CD326^+^ (**Supplementary Figure 3C**) (left), and the frequency of BLIMP-1eGFP^+^ expressing cells within the indicated ASC populations (right), during the time course after isolation. **(C)** Frequency of ASCs expressing BLIMP-1eGFP and given markers as shown in the **Supplementary Figure 3D** from spleen before and after collagenase (Coll) treatment. Populations were gated on CD3^-^ live cells. **(D)** Representative FACS plots (top) and frequencies (left bottom) demonstrating that ASCs can be identified as CD39^+^CD130^+^ and CD39^+^CD81^+^ ASCs before and after Coll treatment. Frequencies of BLIMP-1eGFP^+^ cells within these gates (right bottom). Groups were compared using paired t test **(C, D)**. Data show compilation of 2 independent experiments (n=7 mice). Data show mean ± SEM (*p < 0.05, ***p < 0.001).

In mice, BM ASCs can be identified as BLIMP-1eGFP^+^CD138^+^ or CD19/B220^low/-^CD138^hi^ (**Supplementary Figure 3A**). Recently, it was shown by flow cytometry that ASCs can be identified, in the absence of BLIMP-1, as CD138^+^CD267^+^ (33) (**Supplementary Figure 3A**). While evaluating the stability of the newly identified markers, we observed that CD138 and CD267 expression were down-regulated shortly after ASCs are released from the hypoxic BM environment (**Figure 3A**; **Supplementary Figure 3B**). In contrast, newly identified ASC markers CD39, CD81, CD326, and CD130 showed a stable expression at all tested time points (**Figure 3A** and **Supplementary Figure 3B**). Next, we evaluated optimal marker combinations to mark BM ASCs. For BM ASCs, CD39^+^CD130^+^, CD39^+^CD81^+^, CD39^+^CD326^+^, or CD81^+^CD326^+^ exhibited stable staining with more than 95% of the BLIMP-1eGFP^+^ cells (**Figure 3B** and **Supplementary Figure 3C**).

Isolation of ASCs from non-lymphoid organs is a bottle neck for studying tissue-resident cells. That is, the immune cells need to be released from the tissues by mechanical or enzymatic methods or combinations thereof. In particular, enzymatic digestion affects the detection of plasma cell markers, such as CD138 and CD267 (34). Therefore, we tested whether the newly identified ASC markers would resist to enzymatic digestion. We compared the expression of the markers—CD138, CD267, CD39, CD81, CD326 and CD130—before and after digestion of *prdm1*eGFP spleens with collagenase. This lymphoid organ does not need digestion for ASC retrieval, and therefore, allows for the quantitative comparison of markers stability with or without enzymatic digestion. While CD138 and CD267 disappeared from the cell surface upon collagenase treatment, CD39, CD81, CD326 and CD130 remained clearly detectable (**Figure 3C** and **Supplementary Figures 3D, E**). Thus, CD39 combined with CD81 or CD130 identified tissue resident ASCs with more than 95% of the cells being BLIMP-1eGFP^+^ cells (**Figure 3D**). Of note, collagenase treatments increased ASC retrieval, which was visible with both BLIMP-1eGFP reporter and CD39, CD81, and CD130.

### CD39, CD81, CD130, and CD326 Expression Identifies Human ASCs

**Figure 4:**
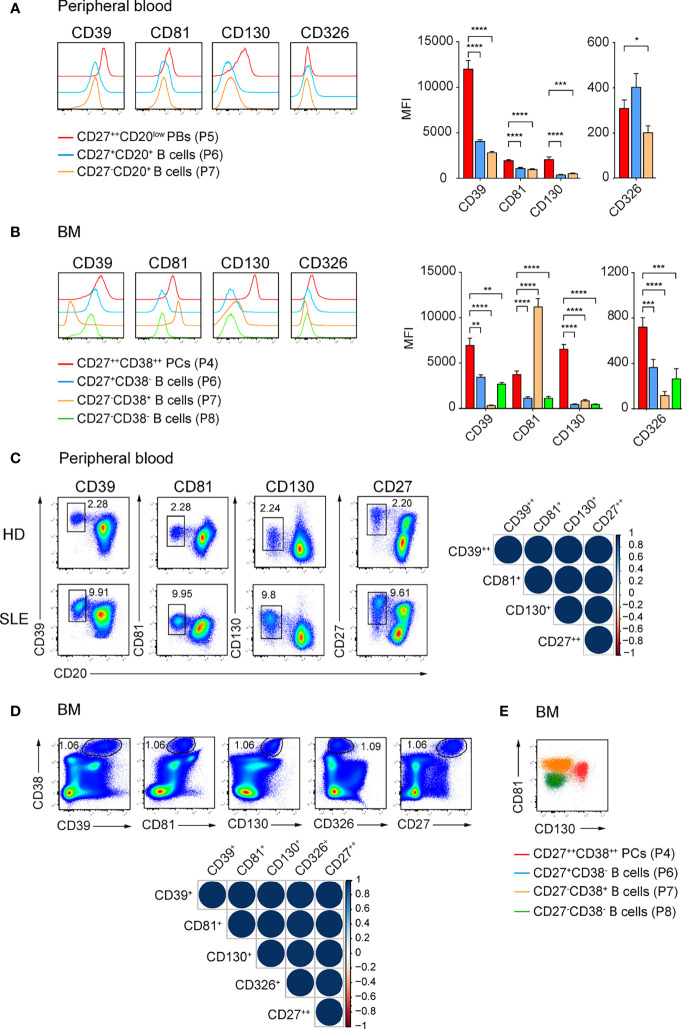
CD39, CD81, and CD130 expression reliably identifies human ASCs. **(A, B)** Representative histograms (left) and graph (right) showing the expression of the indicated surface molecules by ASCs versus other B cell subsets in human peripheral blood **(A)** and BM **(B)**. ASCs and B cell subsets from peripheral blood and BM were gated as shown in **Supplementary Figure 4A, B**, respectively. Groups were compared using two-way ANOVA with the Geisser-Greenhouse correction followed by Dunnett’s multiple comparisons test. **(C, D)** FACS plots showing ASCs identification by the co-expression of CD39, CD81, CD130 or CD27 and CD20 in peripheral blood (C, left) and by the co-expression of CD39, CD81, CD130, CD326 or CD27 and CD38 in BM (D, top). **(C)** CD39^++^CD20^low^, CD81^+^CD20^low^, CD130^+^CD20^low^, and CD27^++^CD20^low^ cells were gated on P4 (**Supplementary Figure 4A**). **(D)** CD38^++^CD39^+^, CD38^++^CD81^+^, CD38^++^CD130^+^, CD38^++^CD326^+^, and CD38^++^CD27^++^ cells were gated on P3 (**Supplementary Figure 4B**). Spearman correlation matrix showing reliable identification of ASCs populations in peripheral blood (C, right) and BM (D, bottom). Blue color coding indicates positive correlation with color intensity and the size of circle reflecting correlation coefficients. **(E)** Representative FACS plot showing the expression CD81 and CD130 by BM CD27^++^CD38^++^ PCs (red), CD27^+^CD38^-^ memory B cells (blue), CD27^-^CD38^+^ (orange) and CD27^-^CD38^-^ (green) immature B cells. Data were a compilation of 12 HDs and 6 SLE for peripheral blood, and 9 HDs for BM. Data show mean ± SEM (^∗^p < 0.05, ^∗∗^p < 0.01, ^∗∗∗^p < 0.001, ^∗∗∗∗^p < 0.0001). P values > 0.05 are not shown.

Our initial findings that CD39, CD81, CD326, and CD130 identify ASCs in mouse led us to examine whether these molecules also identify human ASCs. We isolated mononuclear cells from peripheral blood and BM from heathy donors (HDs) and analyzed the expression of selected molecules by ASCs and other B lineage cells. Particularly, CD27^++^CD20^low^ plasmablasts (PBs), memory CD27^+^CD20^+^, naïve CD27^-^CD20^+^ B cells in peripheral blood, and CD38^++^CD27^++^ PCs, CD27^+^CD38^-^ memory, CD27^-^CD38^+^ and CD27^-^CD38^-^ immature B cells in the BM were analyzed (**Supplementary Figures 4A, B**) (35). CD27^++^CD20^low^ PBs from peripheral blood expressed higher level of CD39, CD81, and CD130 compared with other B cell subsets; and a higher level of CD326 than naïve B cells did (**Figure 4A**). CD38^++^CD27^++^ PCs from BM expressed higher level of CD39, CD130, and CD326 compared with other B cell subsets but lower level of CD81 than CD27^-^CD38^+^ immature B cells did (**Figure 4B**).

We then asked whether the expression of these molecules was sufficient to identify human ASCs. We found similar frequencies of peripheral blood PBs (**Figure 4C**; **Supplementary Figure 4C**) and BM PCs (**Figure 4D**; **Supplementary Figure 4D**) using the newly identify markers or with conventional CD20^low^CD27^++^ and CD38^++^CD27^++^ gating, respectively. Analysis with Spearman correlation matrices showed that the ASC populations identified with new markers correlated with CD27^++^CD20^low^ PBs from peripheral blood (r > 0.97; **Figure 4C**) or with CD38^++^CD27^++^ PCs from BM (r = 1, **Figure 4D**). Most importantly, we verified the expression of these surface molecules in patients with SLE, which have been known to have a higher number of PBs in peripheral blood. CD39, CD81, and CD130 were found on ASCs from peripheral blood of these patients (**Figure 4C**). Next, we evaluated which combination of these markers would be useful to stain PCs in human BM (**Figure 4E**; **Supplementary Figure 4E**). We were able to gate human BM PCs as CD81^+^CD130^+^ cells (**Figure 4E**). We conclude that CD39, CD81, and CD130 expression marks human ASCs from peripheral blood and BM. These surface markers expressed by mouse and human ASCs overcome prior limitations of detection and might candidate as treatment targets of ASCs.

### Microbiota-Derived Signals Downregulate CD81 Expression on ASCs

**Figure 5:**
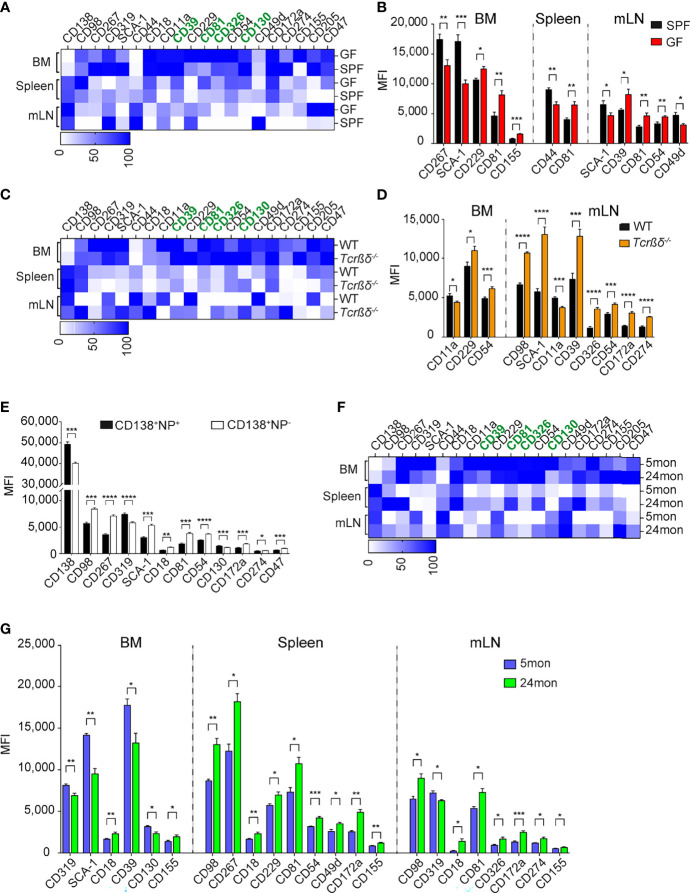
Microbial and T-dependent signals are involved in the regulation of the newly identified markers on ASCs. **(A, C, F)** Heat map showing the expression level of indicated markers by CD138^+^CD39^+^ ASCs from BM, spleen, and mLN of GF versus SPF mice **(A)**, *Tcrβδ^-/-^* versus WT mice **(C)**, and 5 versus 24 months old mice **(F)**. Data are presented as percentages obtained by normalizing the MFI values of each marker among six indicated ASC populations using GraphPad Prism 9. Zero and 100 percent were defined as the smallest and largest mean in each data set, respectively. **(B, D, G)** Expression level (MFI) of indicated markers by CD138^+^CD39^+^ ASCs from BM, spleen, and mLN of GF versus SPF mice **(B)**, *Tcrβδ^-/-^* versus WT mice **(D)**, and 5 versus 24 months old mice **(G)** (markers lacking significant differences are not shown). **(E)** C57BL/6 mice were immunized with 200µg NP-KLH, and splenocytes were analyzed 7 days later. Distinct expression (MFI values) of the indicated markers expressed on NP-reactive versus non-NP-reactive ASCs identified as shown in **Supplementary Figure 5D**. Data show compilation of at least two independent experiments (n=5-9 mice/group). Groups were compared using multiple unpaired t test with Welch correction followed by Holm-Šidák’s multiple comparisons test. Data show mean ± SEM (^∗^p < 0.05, ^∗∗^p < 0.01, ^∗∗∗^p < 0.001, ^∗∗∗∗^p < 0.0001). P values >0.05 are not shown.

The identification of these new and overarching ASC markers may allow new insights into ASC induction and lifestyle. We conducted additional studies to address which signals could promote the expression of the identified markers in ASCs. The level of expression of the several surface markers is organ specific (**Supplementary Figure 5A**). For instance, the transcription factor BLIMP-1 and surface markers, such as CD39, CD81, CD326, or SCA-1 are higher expressed in BM than in spleen and mLN. In contrast, CD138 is higher expressed in spleen and mLN. In addition, splenic ASCs express higher levels of CD98 than those residing in BM and mLN while mLN ASCs express higher levels of CD44 (**Supplementary Figure 5A**). Some ASCs subsets are enriched in certain organs, such as long-lived plasma cells in the BM and microbiota driven ASCs found in the gut-associated tissue, such as the mLN. However, which signals drive the expression of certain molecules on these ASCs is unknown. To delineate a critical pathway of ASC induction and to examine whether CD39, CD81, CD130, and CD326 are expressed by ASCs generated in microbiota-free conditions, we compared the expression of these markers in mice raised in specific pathogen-free (SPF) or in germ-free (GF) conditions. ASCs from GF mice and controls were identified as CD138^+^CD39^+^ cells (**Supplementary Figure 5B**). The frequency of ASCs in BM and spleen were similar in GF and SPF mice (**Supplementary Figure 5B**). As previously described (36), we found a decreased frequency of ASCs in mLN of GF compared to SPF mice, highlighting the importance of microbial signals on ASC generation in mLN (**Supplementary Figure 5B**). We then compared the expression levels of ASCs markers in the two cohorts of mice. Most markers, including CD39, CD130, and CD326, showed similar expression in both conditions (**Figures 5A, B**). In contrast, CD81 was found to be up-regulated on ASCs from GF mice in all analyzed organs (**Figure 5B**; **Supplementary Figure 5C**). We conclude that CD81 expression is downregulated by microbiota-derived signals.

### CD98, SCA-1, CD54, CD172a, and CD274 Expression by ASCs Is Regulated by T Cells

As ASCs can be generated through T-dependent or T-independent pathways, next we examined whether some of the new markers could identify ASCs generated upon canonical T:B cell interaction. To this aim, we analyzed ASCs from T cell-deficient mice (*Tcrβδ* ^-/-^). These mice accumulated less ASCs in BM and more in mLNs than WT mice, while similar frequencies of these cells were found in the spleen (**Supplementary Figure 5D**). The expression of CD39, CD81, CD326, and CD130 by ASCs from T cell-deficient mice was similar or higher than those from WT mice in BM, spleen, and mLN, suggesting that these markers identify ASCs independently of their T-dependent or independent origin (**Figure 5C**). We found similar expression of the newly identified markers in splenic ASCs from WT and T cell-deficient mice, while there were some differences in BM and mLN. CD11a was decreased and CD54 was increased on ASCs from *Tcrβδ* ^-/-^ mice in both BM and mLN compared to those from WT mice (**Figure 5D**; **Supplementary Figure 5E**). Furthermore, we found an increased expression of CD98, SCA-1, CD39, CD326, CD54, CD172a, and CD274 in mLN ASCs from T cell deficient mice compared to WT (**Figure 5D**; **Supplementary Figure 5E**). To further investigate the role of T cell help in the regulation of expression of these molecules on ASCs, we immunized WT mice with 4-hydroxy-3-nitrophenylacetyl keyhole limpet hemocyanin (NP-KLH), a well-established T cell-dependent antigen, and analyzed the expression of ASC markers by non NP- and NP-specific ASCs (**Supplementary Figure 5F**). In agreement with the findings in T cell-deficient mice, non-NP-specific ASCs expressed higher amounts of CD98, SCA-1, CD54, CD172a, and CD274 than NP-specific ASCs (**Figure 5E**; **Supplementary Figure 5G**). Altogether, these data suggest that ASCs generated in the context of T-dependent antigens express lower level of CD98, SCA-1, CD54, CD172a, and CD274.

### ASCs in Aged Naive Mice Are Mainly Generated in a T-Independent Manner

We and others showed that ASCs are accumulated in BM and spleen with age (1, 37). We analyzed the expression of the new markers by ASCs from aged mice, hereafter named, old ASCs in order to identify their origins (37). Flow cytometry analyses confirmed the accumulation of ASCs in BM, spleen, and mLN with aged when CD138 and CD39 were applied to identify ASCs. The increased frequency of ASCs was associated with increase of LAG-3^+^ regulatory ASCs (**Supplementary Figure 5H**) (1). These ASCs were generated independently of T cells as they were also present in T-cell deficient mice (**Supplementary Figure 5I**). Consistent with the data from *Tcrβδ* ^-/-^ and the analysis of non-NP-specific ASCs (**Figures 5D, E**), we found that old ASCs expressed higher levels of a set of four markers, including CD98, CD54, CD172a, and CD274 than ASCs obtained from young animals (**Figures 5F, G**). Collectively, the biased expression of the T independent markers CD98, CD54, CD172a, and CD274 by old ASCs support that ASCs accumulated in old mice share phenotypic characteristics with T-independent ASCs.

### CD39 and CD326 Co-Expression Identifies a BM specific ASC Subpopulation in Lupus Mice

**Figure 6:**
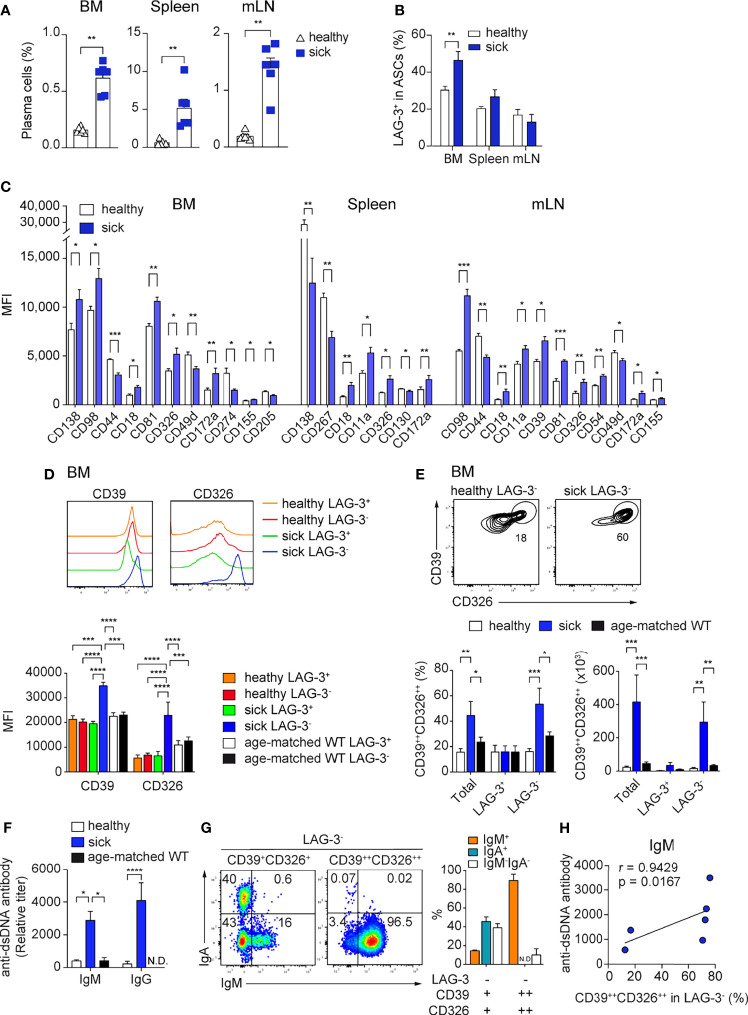
High CD39 and CD326 surface expression identifies a BM specific ASC subpopulation in SLE. *Sle123* mice with proteinuria >100mg/dL **(A–C)** and >300mg/dL **(D–G)** were analyzed. Age-matched WT and healthy mice (2-3 months) without proteinuria were included as controls. **(A, B)** Frequencies of ASCs defined by co-expression of CD138 and CD39 **(A)** and frequencies of LAG-3^+^ cells within ASCs (CD138^+^CD39^+^) of healthy versus sick *Sle123* mice **(B)**. **(C)** Comparison of the expression levels (MFI) of indicated markers on CD138^+^CD39^+^ ASCs from healthy versus sick *Sle123* mice (markers lacking significant differences are not shown). **(D)** Representative histograms (top) and quantification (bottom) of the expression of CD39 and CD326 by LAG-3^+^ and LAG-3^-^ ASCs in BM of healthy and sick *Sle123* and age-matched WT. **(E)** Representative FACS plots (top) showing a subpopulation co-expressing CD39 and CD326 within LAG-3^-^ ASCs in BM of sick *Sle123* compared with healthy controls. The graphs show frequencies (left bottom) of CD39^++^CD326^++^ in total, LAG-3^+^ or LAG-3^-^ ASCs, and numbers (right bottom), in BM of healthy and sick *Sle123* and age-matched WT. **(F)** Anti-dsDNA autoantibody titers from healthy versus sick *Sle123* and age-matched WT mice detected by ELISA. **(G)** Representative FACS plot (left) and graph (right) showing the immunoglobulin isotypes in LAG-3^-^CD39^+^CD326^+^ and LAG-3^-^CD39^++^CD326^++^ from BM of sick *Sle123*. **(H)** Spearman correlation between the frequencies of CD39^++^CD326^++^ within LAG-3^-^ ASCs and anti-dsDNA antibody titer for IgM. Data show compilation of at least two independent experiments (n=6-10 mice/group). Groups were compared using unpaired t test with Welch’s correction **(A, B)**, multiple unpaired t test with Welch correction followed by Holm-Šidák’s multiple comparisons test **(C)**, two-way ANOVA followed by Dunnett’s multiple comparisons test (D-F). Data show mean ± SEM (^∗^p < 0.05, ^∗∗^p < 0.01, ^∗∗∗^p < 0.001, ^∗∗∗∗^p < 0.0001). P values > 0.05 are not shown.

In autoimmunity, pathogenic ASCs produce autoantibodies and pro-inflammatory cytokines (3, 38–41). Thus, is of interest to find molecules that are specifically expressed by pathogenic ASCs, as this would allow their selective targeting. We analyzed the expression of the newly identified ASC markers in the context of lupus using the B6.*Sle123* triple congenic mouse strain with established proteinuria (>100mg/dL, sick). We used B6.*Sle123* mice in which proteinuria was not detectable as controls (healthy). We found that CD39, CD81, and CD130 were highly expressed by ASCs from sick *Sle123* mice (**Supplementary Figure 6A**) and sick *Sle123* mice had an increased frequency of CD138^+^CD39^+^ ASCs in BM, spleen, and mLN compared with the control group (**Figure 6A**). In the BM, this increase was partly associated with enhanced LAG-3^+^ regulatory ASCs (**Figure 6B**).

Next, we evaluated which of the ASC markers could identify disease-associated ASCs in sick *Sle123* mice. CD18, CD326, and CD172a were over-expressed in sick *Sle123* ASCs compared to control ASCs in all analyzed organs (**Figure 6C**; **Supplementary Figure 6B**). In order to fine tune our analysis to pathogenic ASCs, we analyzed the expression of molecules of interest on regulatory LAG-3^+^ ASCs and their counterparts. We found that CD39 and CD326 were over-expressed in BM LAG-3^-^ ASCs compared with LAG-3^+^ ASCs in sick *Sle123* mice, LAG-3^-^ and LAG-3^+^ ASCs in control SLE123 mice and age-matched WT (**Figure 6D**). We did not find similar differences in spleen or mLN (**Supplementary Figure 6C**). Given that CD39 and CD326 were over-expressed only by LAG-3^-^ ASCs in sick *Sle123*, we examined whether their co-expression could identify a new subpopulation of PCs associated with lupus. We found that co-expression of CD39 and CD326 marked an ASC subpopulation (CD39^++^CD326^++^) that accounted for half of total ASCs and the majority of LAG-3^-^ ASCs in sick *Sle123* mice while this ASC population represented less than 20% of total or LAG-3^-^ ASCs in controls. The increased total number of CD39^++^CD326^++^ ASCs in sick *Sle123* mice was mainly associated with increased LAG-3^-^CD39^++^CD326^++^ ASCs (**Figure 6E**). Moreover, the increased frequency of CD39^++^CD326^++^ ASCs in sick *Sle123* was associated with an elevated titers of anti-dsDNA autoantibodies in SLE (**Figure 6F**). We found that CD39^++^CD326^++^LAG-3^-^ were IgM^+^ cells (**Figure 6G**) and the frequency of CD39^++^CD326^++^LAG-3^-^ ASCs correlated with IgM anti-ds-DNA autoantibodies (**Figure 6H**). These cells were not found in spleen, mLN, and kidneys, suggesting that they preferentially reside in the BM and did not correlate with IgG anti-dsDNA autoantibodies in sera of sick *Sle123* mice (**Supplementary Figures 6D, E** **)**.

## Discussion

Herein, we initially identified a set of 12 surface molecules, including CD39, CD229, CD81, CD326, CD54, CD130, CD49d, CD172a, CD274, CD155, CD205, and CD47 that are highly expressed by ASCs, and specifically identify ASCs in naive WT mice as well as in the contexts of immunization and autoimmunity. Among these surface markers, CD39, CD81, CD130, and CD326 provided improved resolution for ASCs identification mice and humans.

BLIMP-1eGFP transgenic mouse models have been instrumental to study ASCs (6, 33, 42–45) but lack utility for human studies. Thus, several approaches have been developed and used to define and quantify ASCs in the absence of the *prdm1*-eGFP allele. ASCs were defined as CD138^+^B220^low^ cells (46) or as CD138^+^SCA-1^+^ cells (45) or as CD138^+^CD267^+^ cells (33). However, while examining the stability of the here reported quartet of markers, in particularly in BMPCs, we observed that CD39, CD81, CD130, and CD326 remained at the cell surface of ASCs for at least 6h after isolation in contrast to CD138 and CD267 disappearing as early as 30 minutes *ex vivo*. Likewise, CD138 has previously been reported to decrease from the surface with use of increasing concentration of sodium azide (45). Importantly, CD138 is often used to follow BM PCs and evaluate their survival (5). Thus, our data suggests that, instead of CD138 and/or CD267, combinations of the newly identified markers may provide advantages for future ASC research. In addition, these new markers are not impaired by enzymatic treatment as it is the case for CD138 and CD267, providing also better solution for tissue resident ASC identification. Furthermore, CD138 and CD267 were downregulated by splenic ASCs from lupus mice (**Figure 6C**).

Translation of findings from mouse ASCs to human ASCs has faced several challenges since some mouse genes do not have direct homologues in humans (6, 47). Therefore, different surface molecules have been used to study mice and humans ASCs (48). Murine ASCs are identified using as CD138^+^ and CD267^+^, while human ASCs have been usually identified as CD27^++^, CD20^low^, and CD38^+^. We found that expression of CD39, CD81 and CD130 can identify ASCs across species, suggesting that these markers are involved in shared and/or overarching functions in ASCs. Therefore, further functional investigation of these molecules may bring important insights into the generation, maintenance, and function of ASCs.

CD39 is the ectonucleoside triphosphate diphosphohydrolase 1 encoded by *ENTPD1* that is responsible for the binding extracellular ATPs and its conversion into adenosine (49–52). Its expression has been associated with several solid tumors, serving as a target in malignant diseases (53, 54). Thus, high expression of CD39 in ASCs may relate to their high metabolic requirements as antibody-producing cells. A very recent study reported that septic conditions can induce the expansion of CD39^hi^ ASCs in spleen together with elevated extracellular adenosine (55). Mice in which only B cells do not express CD39 fail to increase blood adenosine concentration after sepsis. Further analysis showed that CD39 on ASCs plays a key role in converting ATPs into adenosine, thereby promoting immunosuppression activity *via* adenosine-mediated mechanism on macrophages (55). This study corroborates the hypothesis that CD39 is more than a marker of ASCs, but more experiments are needed to elucidate the role of this molecule in the context of ASCs in autoimmunity. CD81, also known as TAPA-1, is a member of the tetraspanin superfamily (TM4SF) that mediates adaptive and innate immune response and is involved in cell adhesion, motility, cell signal transduction and activation, and metastasis. CD81 is expressed by many types of cancer cells (56). In B cells, CD81 is part of the BCR complex that includes CD21 and CD19 and reduces the threshold for B cell activation. It was believed that ASCs no longer had a functional BCR, but this idea has to be reconsidered in light with reports showing that IgM and IgA ASCs still have a functional surface BCR (4, 57, 58). Thus, CD81 may play a role in the activation of ASCs, being therefore also more than just a marker for their identification. CD130 is a signaling molecule associated with cytokine engagement, especially IL-6 and IL-6R (59, 60). Thus, it might be involved in long-term survival of memory PCs in BM, as it is known that IL-6 plays a role in their maintenance (61). CD326 is a transmembrane glycoprotein mediating Ca2^+^-independent homophilic cell-cell adhesion. CD326 was one of the first identified cancer biomarkers (62), which was subsequently used for cancer diagnosis and therapy (63). While additional studies are needed to determine whether these markers play crucial functions in ASCs, the fact that these markers are over-expressed by BM ASCs compared with other ASCs, point them as potential biomarkers for long-lived plasma cells and might serve as therapeutic targets to deplete pathogenic plasma cells.

We found that CD39 is highly expressed by ASCs in mLN from GF and *Tcrβδ* ^-/-^ mice, suggesting that microbial and T cell-derived signals regulate its expression. CD81 is highly expressed by ASCs from mice raised in GF condition in all analyzed organs, suggesting that its expression is regulated by microbial signals. We identified a set five markers, including CD98, SCA-1, CD54, CD172a, and CD274 that are up-regulated by ASCs from T cell deficient mice and down-regulated in NP-specific ASCs upon NP-KLH immunization. These data suggest that canonical T:B cell interaction lead to the production of ASCs expressing low level of these molecules. The current data suggest that their expression could be used as potential biomarkers to classify the T-dependent or -independent origin of ASCs in mouse.

More important, we found that high co-expression of CD39 and CD326 identifies a BM resident ASC subpopulation of LAG-3^-^ that are IgM^+^ and correlates with IgM anti-dsDNA autoantibodies in sera of lupus mice. The identification of this subpopulation is intriguing, because IgM anti-dsDNA autoantibodies have been shown to inhibit glomerular immune-complex deposition, reducing inflammation, in a mouse model of lupus (64). In humans, these antibodies were shown to be negatively associated with lupus nephritis (65). On the other hand, we have previously defined LAG-3^+^ expression PCs as natural occurring regulatory PCs, producing the anti-inflammatory cytokine IL-10 (1). Thus, this data opens the possibility that in autoimmune context, PCs with a regulatory role might also be present in the LAG-3^-^ compartment.

In conclusion, our work suggests that CD39, CD81 CD326, and CD130 represent improved ASC markers in mouse and human. Furthermore, murine ASCs should be identified by co-expression of CD39 with CD81, CD130, or CD326 or co-expression of CD81 with CD130 or CD326. We clearly documented a quartet of molecules that will provide an advance strategy to define and quantify ASCs as well as gain a better understanding ASC biology under various conditions in both mice and humans. Importantly, this study identified an intriguing subpopulation of novel BM resident PCs (CD39^++^CD326^++^) in mice with lupus. Further research is necessary to understand the role of this subpopulation in the disease progression.

## Data Availability Statement

The original contributions presented in the study are included in the article/**Supplementary Material**. Further inquiries can be directed to the corresponding author.

## Ethics Statement

The studies involving human participants were reviewed and approved by Charité Universitätsmedizin Berlin in accordance with Declaration of Helsinki and donors provided written informed consent for participation in the study. The animal study was reviewed and approved by LAGeSo, Berlin.

## Author Contributions

VDD, TD and ACL developed the study concept. VDD, EM, FS, TAL, JR, TH, A-LS, ES, SO, CH, SH, QC, FH, ML, TD, and ACL performed experiments and contributed to project development and wrote of the manuscript. All authors contributed to the article and approved the submitted version.

## Funding

This work was supported by grants from the German Research Foundation LI3540/1-1, Do491/8-1/2 (SPP Immunobone), TRR130/TP24, Do491/10-1, 11-1, and MO2934/1-1.

## Conflict of Interest

The authors declare that the research was conducted in the absence of any commercial or financial relationships that could be construed as a potential conflict of interest.

## Publisher’s Note

All claims expressed in this article are solely those of the authors and do not necessarily represent those of their affiliated organizations, or those of the publisher, the editors and the reviewers. Any product that may be evaluated in this article, or claim that may be made by its manufacturer, is not guaranteed or endorsed by the publisher.

## Supplementary Material

Supplementary Figure 1 ASCs from naive mice express CD39, CD229, CD81, CD326, CD54, CD130, CD49d, CD172a, CD274, CD155, CD205, and CD47. Analyses were performed with naive *prdm1*eGFP mice **(A)** Gating strategy for spleen ASCs CD138^+^BLIMP-1eGFP^+^ (P5), B cells CD19^+^CD138^-^BLIMP-1eGFP^-^ (P6) and non-B cells CD19^-^CD138^-^BLIMP-1eGFP^-^ (P7). **(B, C)** Graphs showing cell frequencies expressing indicated markers by CD138^+^BLIMP-1eGFP^+^ASCs, CD19^+^CD138^-^BLIMP-1eGFP^-^ B cells, and CD19^-^CD138^-^BLIMP-1eGFP^-^ cells from spleen **(B)** and mLN **(C)**. Populations were gated as shown in **Supplementary Panel 1A**. Groups were compared using two-way ANOVA with the Geisser-Greenhouse correction followed by Dunnett’s multiple comparisons test. Data were compiled from two independent experiments (n=6 mice). Data show mean ± SEM (^∗∗^p < 0.01, ^∗∗∗^p < 0.001, ^∗∗∗∗^p < 0.0001). P values > 0.05 are not shown. **(D, E)** Representative FACS plots showing the co-expression of BLIMP-1eGFP with the indicated marker on splenic **(D)** and mLN **(E)** ASCs from naive *prdm1*eGFP mice. The populations were gated on live cells. Results are representative of two independent experiments (n=6 mice).

Supplementary Figure 2 Surface expression of CD229, CD54, CD49d, CD172a, CD274, CD155, CD205, and CD47 identifies ASCs in naive mice. Representative FACS plots showing the co-expression of CD138 with indicated marker in BM, spleen, and mLN from naive *prdm1*eGFP mice. The populations were gated on live cells. Results are representative of two independent experiments (n=6 mice).

Supplementary Figure 3 CD39, CD81, CD130, and CD326 expression is stable and provides improved resolution for ASC identification. Analyses were performed with naive *prdm1*eGFP mice. **(A)** FACS plots (left) show ASC identification and corresponding frequencies of ASCs derived from BM (populations gated on CD3^-^ live cells). **(B, C)** representative FACS plots showing different ways of ASC identification at indicated time points after BM single cell suspension preparation (populations were gated on CD3^-^ live cells). **(D)** FACS plots showing ASC frequencies defined by co-expression of BLIMP-1eGFP and indicated molecule isolated without or with collagenase (Coll) digestion (populations were gated on CD3^-^ live cells). **(E)** Histograms and graphs depicting the expression of the indicated markers by BLIMP-1eGFP^+^CD39^+^ ASCs prepared without or with Coll treatment of spleen as shown in **Supplementary Panel 3D**. Group comparison was performed by paired t test. Data compiled from two independent experiments (n = 7 mice).

Supplementary Figure 4 CD39, CD81, and CD130 expression reliably identifies human antibody secreting cells. **(A, B)** Gating strategy identifying ASCs and B cell subsets in human peripheral blood **(A)** and BM **(B)**. In peripheral blood, ASCs were identified as CD27^++^CD20^low^ (P5), memory B cells as CD27^+^CD20^+^ (P6) and naive B cells as CD27^-^CD20^+^ (P7). In BM, ASCs were CD38^++^CD27^++^ (P4) and B cell subsets such as CD27^+^CD38^-^ (P6), CD27^-^CD38^+^ (P7), CD27^-^CD38^-^ (P8), The BIN channel included CD3, CD14 and live/dead dye. **(C, D)** Frequency of peripheral blood PBs **(C)** and BM PCs **(D)** gated as in **Figure 4C** and **4D**, respectively. **(E)** Representative FACS plot showing the expression of indicated molecules by BM CD27^++^CD38^++^ PCs (red), CD27^+^CD38^-^ memory B cells (blue), CD27^-^CD38^+^ (orange) and CD27^-^CD38^-^ (green) immature B cells. Data show compilation of 12 and 9 donors for peripheral blood and BM, respectively.

Supplementary Figure 5 Microbial and T-dependent signals are involved in the regulation of the newly identified markers on ASCs. **(A)** Expression level (MFI) of the indicated markers by CD138^+^BLIMP-1eGFP^+^ ASCs from BM, spleen, and mLN from naive *prdm1*eGFP mice. Groups were compared using two-way ANOVA with Geisser-Greenhouse correction followed by Tukey’s multiple comparisons test. **(B, D)** Representative FACS plots (left) and quantification (right) showing frequencies of ASCs co-expressing CD138 and CD39 from GF versus SPF **(B)** and *Tcrβδ^-/-^* versus WT mice **(D)**. The populations were gated on live cells. **(C, E)** Histograms showing the expression level of indicated markers by CD138^+^CD39^+^ ASCs from BM, spleen and mLN of GF compared with SPF mice **(C)**, and from BM and mLN of *Tcrβδ^-/-^* compared with WT mice **(E)**. **(F)** Gating strategy (top) identifying CD19^+^CD138^-^ B cells (P5), non-NP-reactive (P6) and NP-reactive CD138^+^ ASCs (P7). The graph (bottom) shows the frequency of NP-reactive versus non-NP-reactive within CD138^+^ ASCs. **(G)** Heat map of the expression level of the indicated markers by CD19^+^CD138^-^ B cells, NP-reactive and non-NP-reactive CD138^+^ ASCs that were identified as in Supplementary . Data were calculated as described in **Figure 5A, 5C** and **5F**. **(H, I)** Representative FACS plots and graph showing the frequency of LAG-3^+^ within CD138^+^CD39^+^ ASCs from 5 versus 24 months old WT mice **(F)** and *Tcrβδ^-/-^* versus WT mice **(G)**. Groups were compared using unpaired t test with Welch’s correction for B, C, D, F and G. Data show compilation of at least two independent experiments (n=5-9 mice/group). Data show mean ± SEM (^∗^p < 0.05, ^∗∗^p < 0.01, ^∗∗∗^p < 0.001, ^∗∗∗∗^p < 0.0001). P values >0.05 are not shown.

Supplementary Figure 6 High CD39 and CD326 surface expression identifies a BM specific ASC subpopulation in SLE. **(A)** Representative FACS plots showing the expression of CD138, CD39, CD81, and CD130 by ASCs from BM, spleen, and mLN from sick *Sle123* mice. The populations were gated on live cells. **(B)** Heat map shows the expression level of indicated markers by CD138^+^CD39^+^ ASCs from BM, spleen, and mLN of healthy and sick *Sle123* mice. The data were obtained as described in **Figure 5A, 5C** and **5F**. **(C)** Quantification of the expression of CD39 and CD326 by LAG-3^+^ and LAG-3^-^ ASCs in spleen (left) and mLN (right) of healthy and sick *Sle123*. Groups were compared using two-way ANOVA followed by Dunnett’s multiple comparisons test. The MFI values are shown in the graph. **(D)** Representative FACS plots showing no subpopulation co-expressing high levels of CD39 and CD326 within LAG-3^-^ ASCs in spleen, mLN, and kidney of sick *Sle123*. **(E)** Spearman correlation between the frequencies of CD39^++^CD326^++^ within LAG-3^-^ ASCs and anti-dsDNA antibody titer for IgG. Data show compilation of two independent experiments (n=6 mice/group). Data show mean ± SEM (^∗∗^p < 0.01, ^∗∗∗∗^p < 0.0001).
