# Supplementary material for: CD39 and CD326 Are Bona Fide Markers of Murine and Human Plasma Cells and Identify a Bone Marrow Specific Plasma Cell Subpopulation in Lupus
Source: Front Immunol. 2022 Apr 8;13:873217. doi: 10.3389/fimmu.2022.873217 (PMC9024045; doi:10.3389/fimmu.2022.873217)
Supplement: Supplementary Figure 1 — ASCs from naive mice express CD39, CD229, CD81, CD326, CD54, CD130, CD49d, CD172a, CD274, CD155, CD205, and CD47. Analyses were performed with naive prdm1eGFP mice (A) Gating strategy for spleen ASCs CD138+BLIMP-1eGFP+ (P5), B cells CD19+CD138-BLIMP-1eGFP- (P6) and non-B cells CD19-CD138-BLIMP-1eGFP- (P7). (B, C) Graphs showing cell frequencies expressing indicated markers by CD138+BLIMP-1eGFP+ASCs, CD19+CD138-BLIMP-1eGFP- B cells, and CD19-CD138-BLIMP-1eGFP- cells from spleen (B) and mLN (C). Populations were gated as shown in Supplementary Panel 1A. Groups were compared using two-way ANOVA with the Geisser-Greenhouse correction followed by Dunnett’s multiple comparisons test. Data were compiled from two independent experiments (n=6 mice). Data show mean ± SEM (∗∗p < 0.01, ∗∗∗p < 0.001, ∗∗∗∗p < 0.0001). P values > 0.05 are not shown. (D, E) Representative FACS plots showing the co-expression of BLIMP-1eGFP with the indicated marker on splenic (D) and mLN (E) ASCs from naive prdm1eGFP mice. The populations were gated on live cells. Results are representative of two independent experiments (n=6 mice). [file DataSheet_1.pdf]

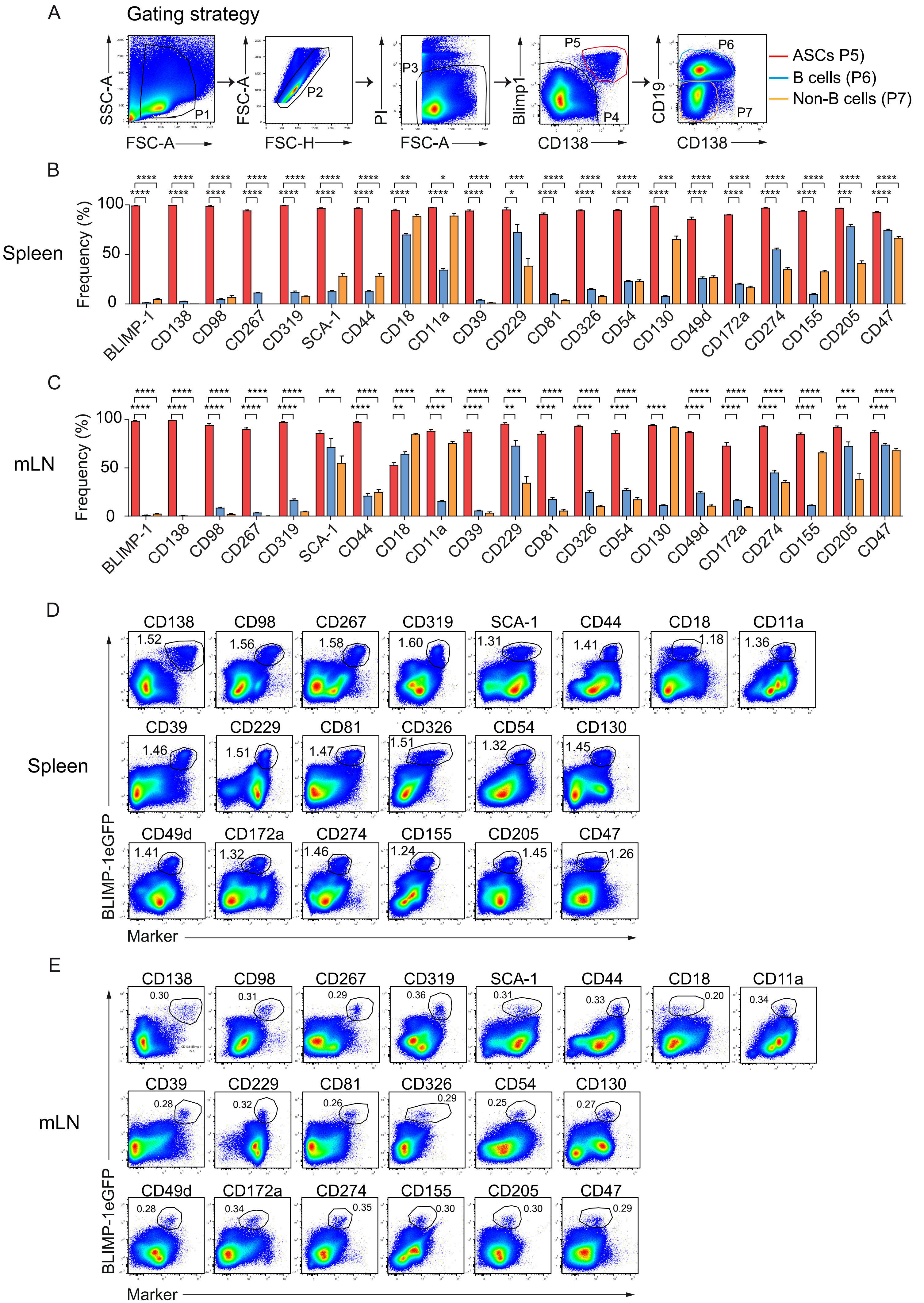

Supplementary Figure 1

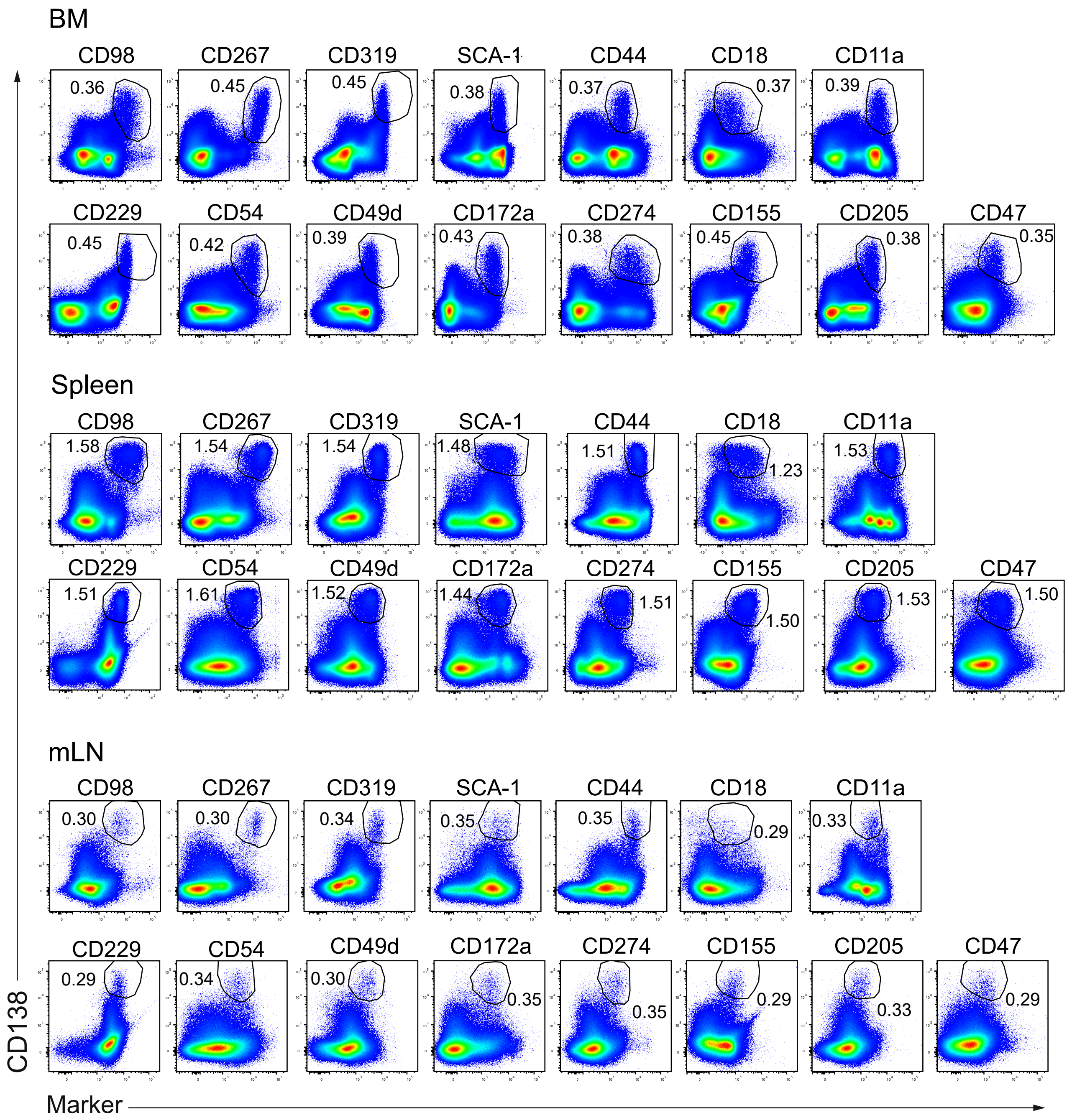

Supplementary Figure 2

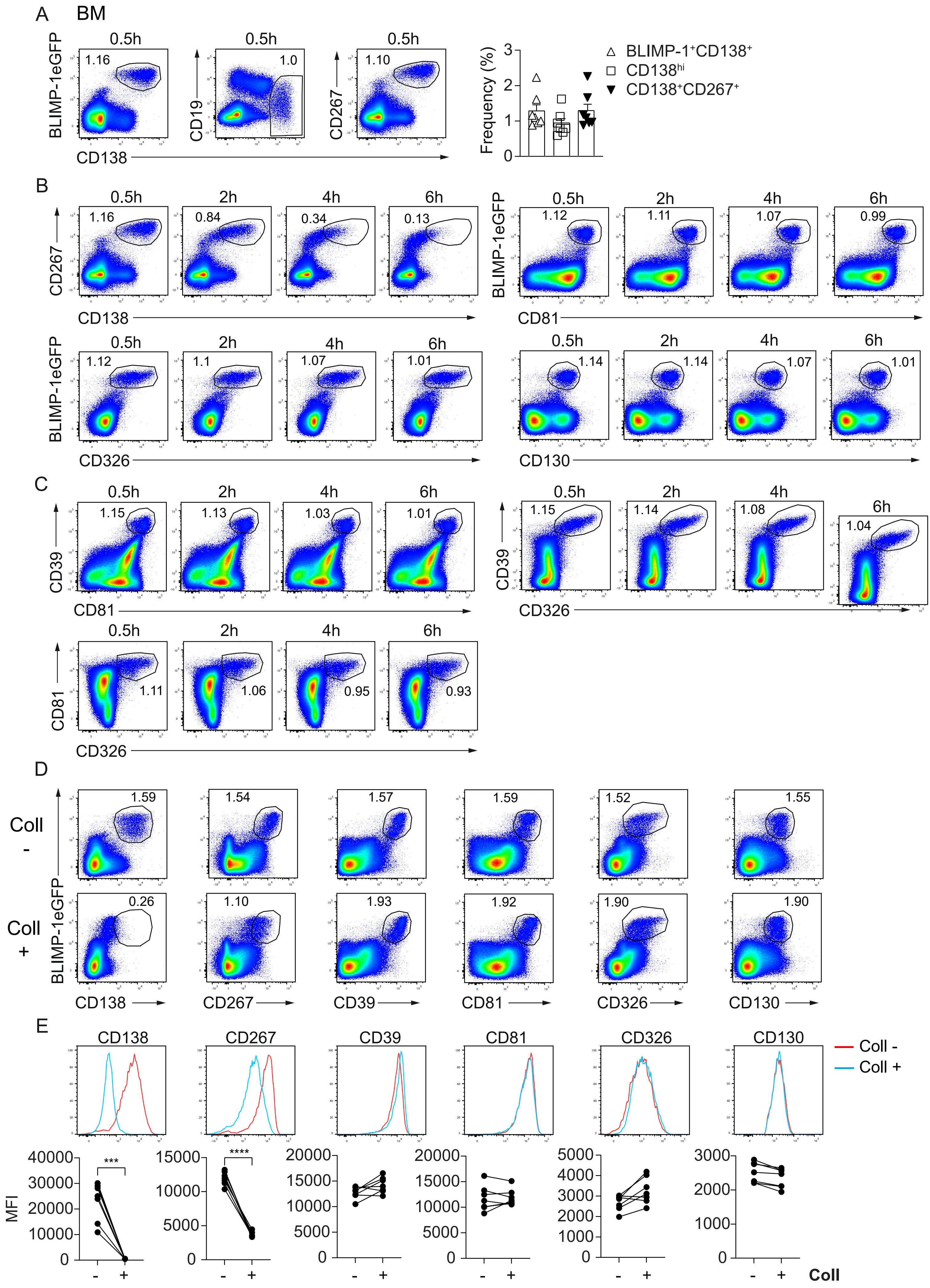

Supplementary Figure 3

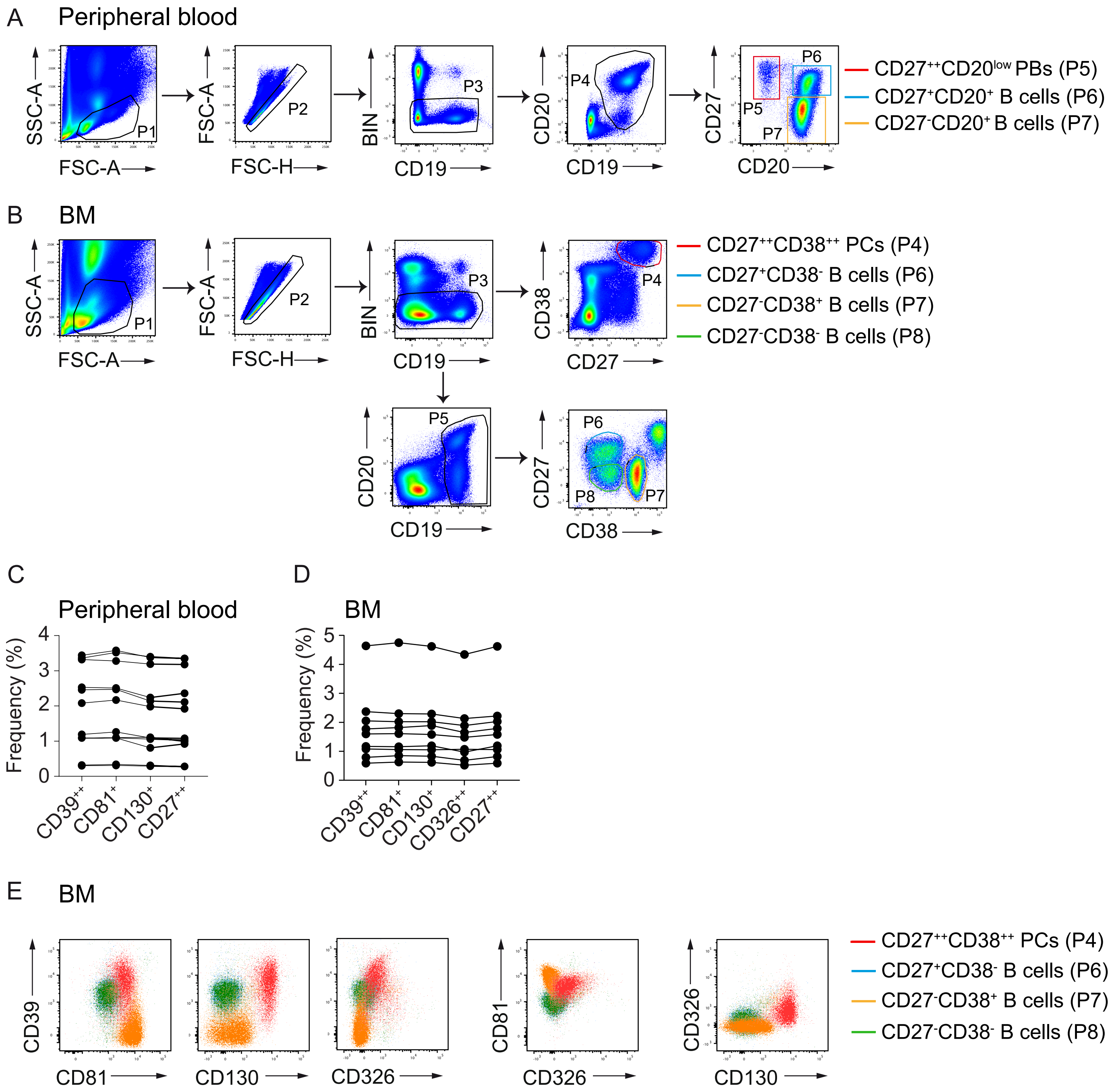

Supplementary Figure 4

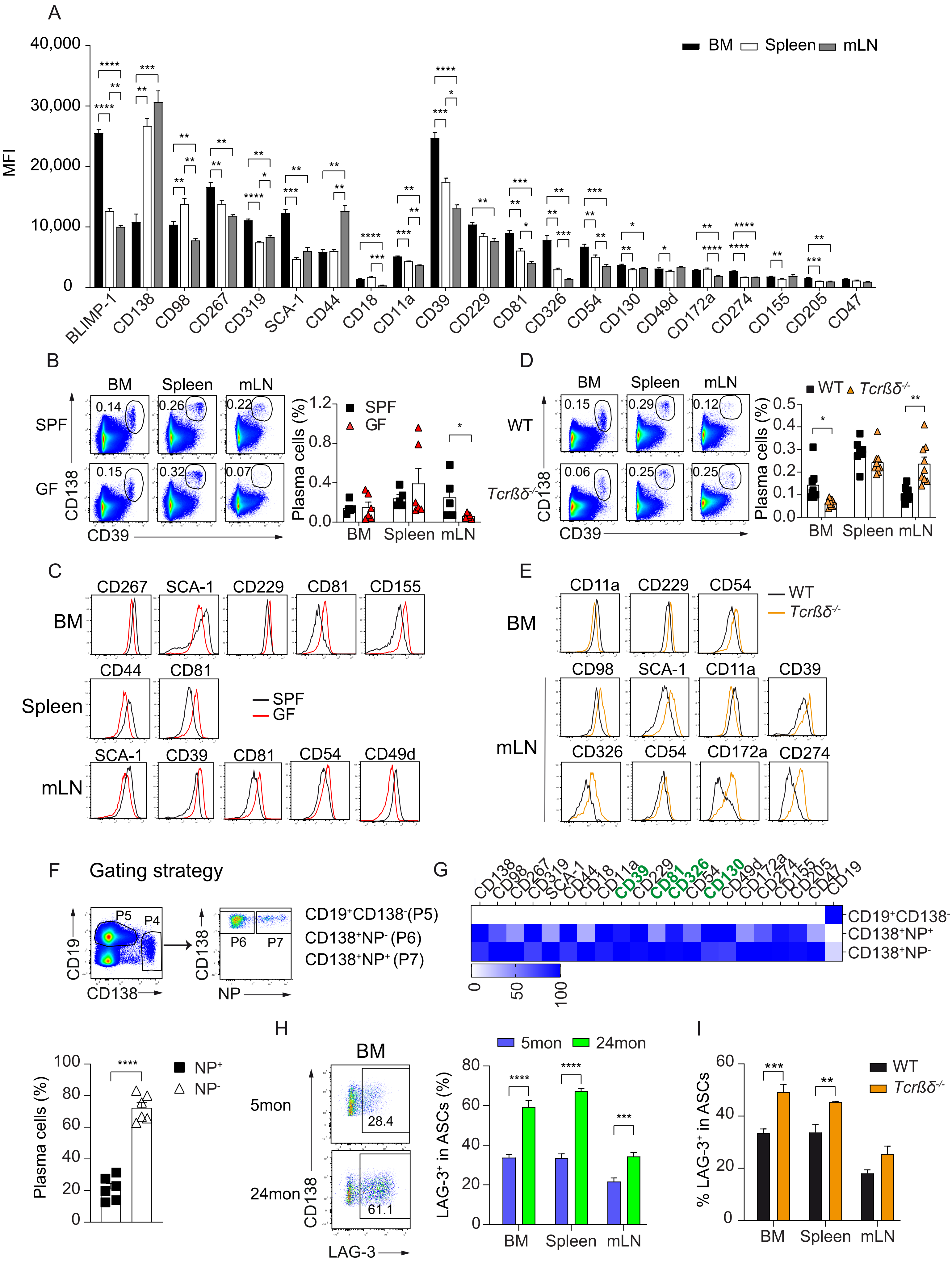

Supplementary Figure 5

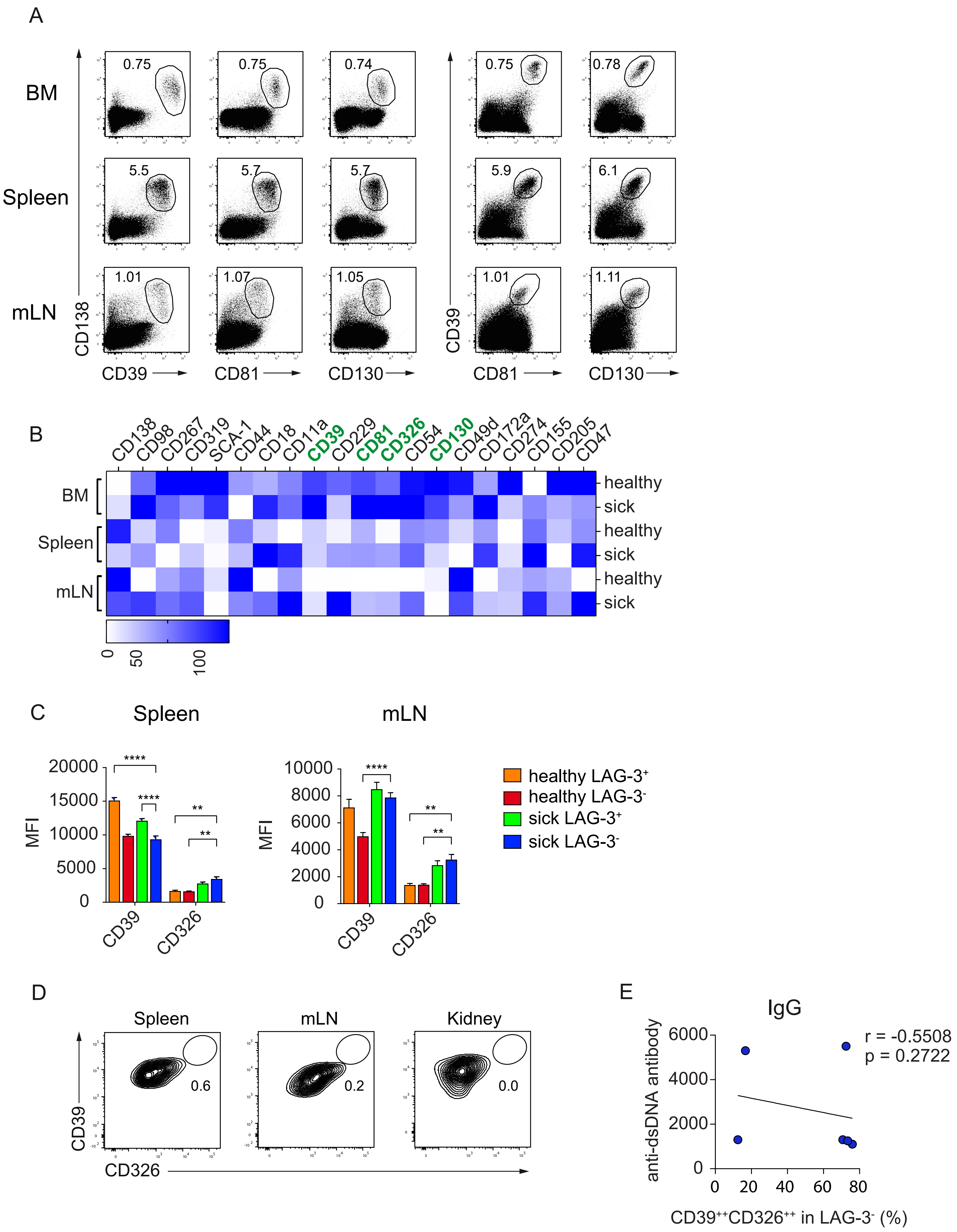

Supplementary Figure 6
